# Does statistics anxiety impact academic dishonesty? Academic challenges in the age of distance learning

**DOI:** 10.1007/s40979-022-00117-w

**Published:** 2022-10-03

**Authors:** Yovav Eshet, Pnina Steinberger, Keren Grinautsky

**Affiliations:** 1 Interdisciplinary Studies, Zefat Academic College, 11 Jerusalem St, Zefat, Israel; 2 Orot Israel College of Education, 3 Steinman St, Rehovot, 76110 Israel; 3 Faculty of Behavioral Sciences, Zefat Academic College, Zefat, Israel

**Keywords:** Academic dishonesty, Statistics anxiety, Personality traits, Learning Environment, Motivation

## Abstract

This study discusses the mediating role of statistics anxiety and motivation in the relationship comprising academic dishonesty, personality traits, and previous academic achievements in three different learning environments (Face to Face -F2F, Planned Online Environment – POE, and Emergency Remote Teaching – ERT). Self-determination theory (SDT) provides a broad psychological framework for these phenomena. Data were collected from 649 bachelor-degree students in the Social Sciences in five Israeli academic institutions. Structural equation modelling was employed to investigate the research variables’ relationships. Findings indicate that statistics anxiety mediates the relationship between personality traits and academic dishonesty in the POE and the ERT learning environments. Findings also indicate the relationship between students’ achievements and academic dishonesty, but only in the ERT learning environment. In contrast, motivation mediates the relationship between students’ achievements and statistics anxiety only in the POE learning environment. This study unveils that learning environments determine the mediating role of statistical anxiety. In digital learning environments (POE, ERT), mediation has been found between students’ personality traits and academic dishonesty. No similar parallel mediation could be established in the physical learning environment, F2F. Thus, we conclude that online courses should be designed according to student-centred approaches.

## Introduction

The COVID-19 pandemic is a global concern affecting Higher Education Institutions (Reedy et al. 2021). Academic institutions worldwide were compelled to postpone or cancel presential lectures and move to distance online teaching (Elsalem et al. 2021). This has affected different educational aspects (Gamage et al. 2020), including academic dishonesty (AD) (Turner et al. 2022). Research has revealed that academic misconduct, like AD, increased dramatically worldwide (Erguvan 2021). AD poses a severe threat, undermining the educational system’s integrity (Miller 2019). Furthermore, AD has both moral and practical implications (Bacon et al. 2020), as students’ ethical behaviour transfers over into the job force (Walsh et al. 2021). Thus, professional education shall also focus on the ethical formation (Guerrero-Dib et al. 2020). Recent research (Etgar et al. 2019) has revealed the pivotal role of motivation in students’ disposition to AD. According to Self-determination theory (SDT) by Ryan and Deci (2008, 2020), motivation results from either internal or external incentives, which indicates the domain of a self-initiated activity prompted by some external factor (Locquiao and Ives 2020). SDT provides a broad psychological framework for understanding motivation for AD (Krou et al. 2021).

In addition, knowledge of statistics has been recognized as mandatory in academic education (Trassi et al. 2022) thus; current academic training includes compulsory introductory statistics courses. Occasionally, some students associate these with high anxiety levels (O’Bryant et al. 2021). For some students experiencing Statistics anxiety (SA), this assignment has a negative impact on their academic experiences (Trassi et al. 2022). Previous research on undergraduate social sciences students (Steinberger et al. 2021) unveiled that students’ anxiety toward statistics negatively influences learning and academic performance. Moreover, anxiety and inappropriate academic behaviours are related (Zhang et al. 2020). In addition, (Tindall et al. 2021) found that negative emotions influence students’ propensity to engage in unethical conduct like plagiarism.

Likewise, research has shown a significant interrelation among attitudes toward statistics, anxiety, and performance, which are determined by students’ prior statistics or mathematics education (Peiró-Signes et al. 2021). Scholarly review literature (Cui et al. 2019; Chiang et al. 2022) has indicated that dispositional character and person-related circumstances determine statistics anxiety (SA). Furthermore, research dealing with the influence of SA on student academic performance is vast (O’Bryant et al. 2021), including factors which predict AD (Roe 2022).

Yet, studies on SA, AD and pandemic circumstances are scanty (Steinberger et al. 2021). Our research fills this gap by examining the relationship comprising: AD, SA, personality traits, and motivation in undergraduate students in the social sciences taking an Introduction to Statistics compulsory course in different learning environments (Face to Face – F2F, Planned Online Learning - POE and Emergency Remote Teaching – ERT). Understanding AD’s profile and likelihood is key to personalising academic interventions meant to discourage and reduce it and SA manifesting in different learning environments. Furthermore, our research enlightens the mediating roles SA and motivation play in the relationship comprising personality traits, previous achievements, and AD. Thus, the main research question is: To what extent does the relationship among SA, personality traits, previous achievements, and motivation affect AD in the different learning environments (F2F, POE, ERT)?

## Theoretical background

### Academic dishonesty

Effective learning and teaching are key research topics in higher education (Steinberger et al. 2021). Academic integrity is a *desideratum* for quality education (Ozoliņa and Bēriņa 2021). Quality education is unattainable without respect for academic integrity (Artiukhov and Liuta 2017), and without maintaining quality educational process standards in the educational (Kudeikina et al. 2022). Understanding this has contributed to expanding scholarly knowledge on academic integrity and preventing AD (Parnther 2020). Whereas academic integrity refers to trustful, respectful, fair, and responsible behaviours (Sefcik et al. 2020), AD refers to offences that include: cheating, plagiarism, fabrication, and facilitation (Etgar et al. 2019). Studies have shown the omnipresence of AD as a normalized student behaviour (Krou et al. 2021; Chiang et al. 2022; Christensen Hughes and Eaton 2022) and that most students engage in AD at some point in their studies (Peled et al. 2019). Furthermore, other studies have indicated that AD is more likely to prevail among certain populations (Hensley et al. 2013). For example, business students have a greater propensity to cheat than non-business students (McCabe et al. 2006).

### Statistics anxiety

Statistical literacy has become an essential skill for higher education students for their academic and future professional practice (Berndt et al. 2021), including in business programs (Vaziri et al. 2022). Yet, studies have revealed that students experience problems with learning, understanding, and using basic statistical notions. Statistics anxiety (SA) is one of the most common phenomena following this (Murtonen 2015). SA refers to a negative emotional state or attitude provoked by any form of contact with statistically related content (O’Bryant et al. 2021). Hence, it often interferes with teaching-learning quantitative material. According to a research literature review (Cui et al. 2019), SA’s antecedents are: (a) Dispositional factors (personality traits), (b) personal factors (previous academic achievement, motivation) and (c) situational factors (attitudes connected to statistics).

The SA six-factor model is a largely acknowledged approach (Levpušček and Cukon 2020), identifying six elements informing SA, which is integrated into the common SA rating scale (STARS) by Cruise et al. (1985). The above encompasses anxious feelings and learners’ attitudes towards statistics: Interpretation anxiety – the anxiety following the need to interpret different statistical data; Test and class anxiety – the anxiety manifesting while attending statistics courses and taking statistics tests; Fear of asking for help – the anxiety manifesting while requesting assistance to understand statistics; Computational self-concept – an individual’s perception of his mathematical abilities for learning statistics; Worth of statistics – the significance and relevance of learning statistics, and Fear of statistics teachers – students’ perceptions of statistics teachers.

### Personality traits

The Five-Factor Model (FFM) by McCrae and Costa (1987) is an acknowledged psychological tool for theoretically evaluating and measuring personality traits (Dimitriadis et al. 2017). The FFM was neither designed to identify nor measure ethical conduct (Sleep et al. 2021). Nonetheless, research has shown that traits are crucial for understanding students’ disposition to engage in AD (Peled et al. 2019). The FFM divides personality into five different traits: Openness to experience, which expresses love for art, adventure, atypical ideas, and imagination; Conscientiousness, which refers to the tendency to exhibit self-discipline and act dutifully; Extraversion, which is intimately related to engagement with the outer world and often characterizes individuals, who are perceived as fully energetic; Agreeableness, which is associated with the value of getting along with others, individuals possessing this latter trait are often considerate, kind, generous, trusting, helpful, optimistic; and Neuroticism (or Emotional Instability), which is associated to the tendency to be subject to negative emotions such as anger, anxiety, stress, and depression. Research has shown that FFM significantly impacts SA (Steinberger et al. 2021). For example, openness to experience and agreeableness correlate negatively with SA, neuroticism and extraversion positively correlate with SA. Conscientiousness does not correlate with it (Cui et al. 2019). This pioneering research may clarify the above and open the road to developing positive educational outcomes and interventions by enlightening the relationship comprising FFM, AD, and SA in the different learning environments (F2F, POE & ERT).

## Academic dishonesty, statistics anxiety, and personality traits

Research on AD has repeatedly employed the FFM (Eshet et al. 2014). It has been revealed that personality determines cheating behaviour due to its impact on personal beliefs, one’s attitude towards learning and studying, and goal achievement approach (Malesky et al. 2022). For example, negative affect predicts plagiarism (Tindall et al. 2021). Furthermore, personality traits are also associated with SA (Chew and Dillon 2014). Previous research on the relationship between personality traits, AD and SA, has suggested that: Students scoring high on openness to experience, who are interested in learning and curious, tend to disapprove of AD (Lee et al. 2020) and exhibit lower anxiety levels (Steinberger et al. 2021). Students scoring high in conscientiousness, with a high propensity to follow the rules, exhibit a low cheating propensity (Giluk and Postlethwaite 2015) and are unrelated to SA (Chew and Dillon 2014). Students scoring high on extraversion are often assertive and prone to cheat (Malesky et al. 2022) and are also positively associated with some components of SA (Agbaria and Mokh 2021). Students scoring high on agreeableness have a significantly negative correlation to AD (Malesky et al. 2022) and SA (Cui et al. 2019). Students scoring high on neuroticism (those scoring low on emotional stability) positively correlate with AD (Muntada 2013) and SA. Thus, we posit:

### H1:

Statistics Anxiety will mediate the relationship between Students’ Personality Traits and Academic Dishonesty

## Motivational orientation

Motivation can psychologically strengthen and stimulate students’ learning processes and activities (Becerra and Almendra 2020). Accordingly, it predicts academic performance (Tonguç and Ozaydın Ozkara 2020; Zalts et al. 2021) as it explains one’s intentional behaviours (Shi et al. 2021). Furthermore, motivation is a substantial factor in conditioning anxiety (Luo et al. 2020). For example, students having low mathematical proficiency will display higher anxiety rates (Faber and Drexler 2019) and negative attitudes towards statistics (Bromage et al. 2021). According to Self-Determination Theory (SDT) by Deci and Ryan (2008), motivation can either be intrinsic or extrinsic. Intrinsic motivation refers to the willingness to engage in educational activities based on inherent characteristics (genuine interest and enjoyment). Conversely, extrinsic motivation points to one’s incentives for doing something due to external outcomes or rewards. Intrinsic motivation is positively associated with academic success, performance, and self-confidence (Foutz et al. 2021). Studies have pointed out that intrinsic motivation positively impacts self-confidence and responsibility, while extrinsic motivation relates to incompatible behaviours such as anxiety and indifference towards responsibility (Lavasani et al. 2014). Students scoring high on extrinsic motivation are driven by grades, class rank, and earnings (Zalts et al. 2021). Furthermore, motivation and FFM are positively related to academic performance. Research suggests that according to the different personality traits, there are different motivational orientations (Arniatika 2020). For instance, consciousness and openness to experiences correlate with intrinsic motivation; neuroticism correlates with extrinsic motivation (Müller et al. 2006). Thus, we posit:

### H2

Students’ Motivation will mediate the relationship between Students’ Personality Traits and Statistics Anxiety.

## Previous achievement, statistics anxiety, and academic dishonesty

Previous academic achievement predicts future academic outcomes (Hensley et al. 2013) and success in statistics courses (Sorge and Schau 2002). A myriad of research has explored the cognitive and affective factors that influence students’ performance in statistics. According to widespread conceptions, poor achievements is strongly connected to academic misconduct (Koscielniak and Bojanowska 2019). For example, prior research has found that previous academic performance and SA strongly correlate (Siew et al. 2019; Steinberger et al. 2021). For example, some students experience SA due to their lack of mathematical knowledge (Onwuegbuzie and Wilson 2003). Therefore, linking anxiety to performance leads students to higher procrastination rates and avoid statistics-related tasks (Onwuegbuzie and Wilson 2003). This leads to avoidance behaviour (Hong, Tsai, & Tai, 2021) as a form of compensations strategy to better results (Koscielniak and Bojanowska 2019), inducing AD. Other research has emphasized that ethical value behaviour can be ascribed to motivation, learning strategies, and students’ previous achievements (Koscielniak and Bojanowska 2019), including AD. Thus, we posit:

### H3

Statistics Anxiety will mediate the relationship between Students’ Previous Achievements and Academic Dishonesty.

## Academic dishonesty since the Covid-19 in different learning environments

Practices of AD before the COVID-19 pandemic have been diverse (Gamage et al. 2020). Scholarly studies before the latter’s outburst could determine that technology’s proliferation has changed the nature of AD offences (Meiring 2019). Yet, knowledge of the impact of these since Covid-19 is still scanty. Online instruction has grown exponentially since the pandemics’ outburst, thus altering educational practices’ nature and delivery for years to come (Li and Lalani 2020). Furthermore, responses to the pandemic strengthen the active dimension of education (Crawford 2020), e.g., active learning, independent and critical thinking, individual exploration and participatory development (Armellini et al. 2021). Students adapt to different learning environments in this context, often incompatible with academic attendance obligations (Butler-Henderson and Crawford 2020). Scholars have added that the diversity of educational situations and the implementation of heterogeneous learning technologies have led to different educational theories about how technology impacts education (Venn et al. 2020). Hence, the importance of meticulously clarifying how digital integrity has become a crucial 21st-century skill impacting students’ ability to evaluate, handle, and share knowledge ethically and successfully (Miller 2019). Notably, there is a broad scholarly consensus on the pedagogical divergences between online and face-to-face teaching. The mere application of traditional educational approaches to online settings has been shown to be ineffective due to the tensions it often creates (Badiozaman 2021). Contextualizing the above phenomenon is mandatory to understand it fully.

The learning environments include psychological, pedagogical, and social features influencing students’ achievement (Helms 2014). The last decades have witnessed a transformation in the learning environments. These changes further enabled the implementation of innovative pedagogical approaches entwined with modern technology (Valtonen et al. 2021), like the integration of information & communication technology (ICT) teacher or student-centred approaches (Mesny et al. 2021). Educators frame and decide on the course’s structure, content, and educational process (Greenberg et al. 2007). Furthermore, technological advancement has led to digitalized - entirely online (planned online environment- POE) or hybrid modules of traditional face to face (F2F) education. Consequently, this has raised concerns about AD and its different ways of cheating (Ikram and Rabbani 2021). In addition, the Covid-19 pandemic impelled education to transform into online delivery (Turnbull et al. 2021), leading to unplanned online teaching and learning formats (Lowenthal et al. 2020), coined as emergency remote teaching - ERT (Hodges et al. 2020). This has further led education to new challenges (Whalen 2020). Previous scholarly research has shown that students’ learning experience and performance differ according to learning environments (Mørk et al. 2020; Maqableh and Alia 2021), including course enrolment and delivery methods: F2F, POE, and ERT. For instance, instructive intensity, disorganization, and oppression likely raise SA levels. Additionally, the learning environment and teachers’ interactions influence students’ motivation (du Rocher 2020). Studies focusing on statistics learning have compared results obtained from POE and F2F statistics courses. POE instruction is less effective than F2F, as it allows learners to be more concretely exposed to their educator’s attitudes and concerns. As a result, performance in POE settings is lower than in F2F ones (Cui et al. 2019). Thus, we posit:

### H4

There will be differences between learning environments in the relationship comprising Statistics Anxiety, Personality Traits, Motivation, Academic Dishonesty, and Previous Academic Achievement.

## Research model

**Fig. 1:**
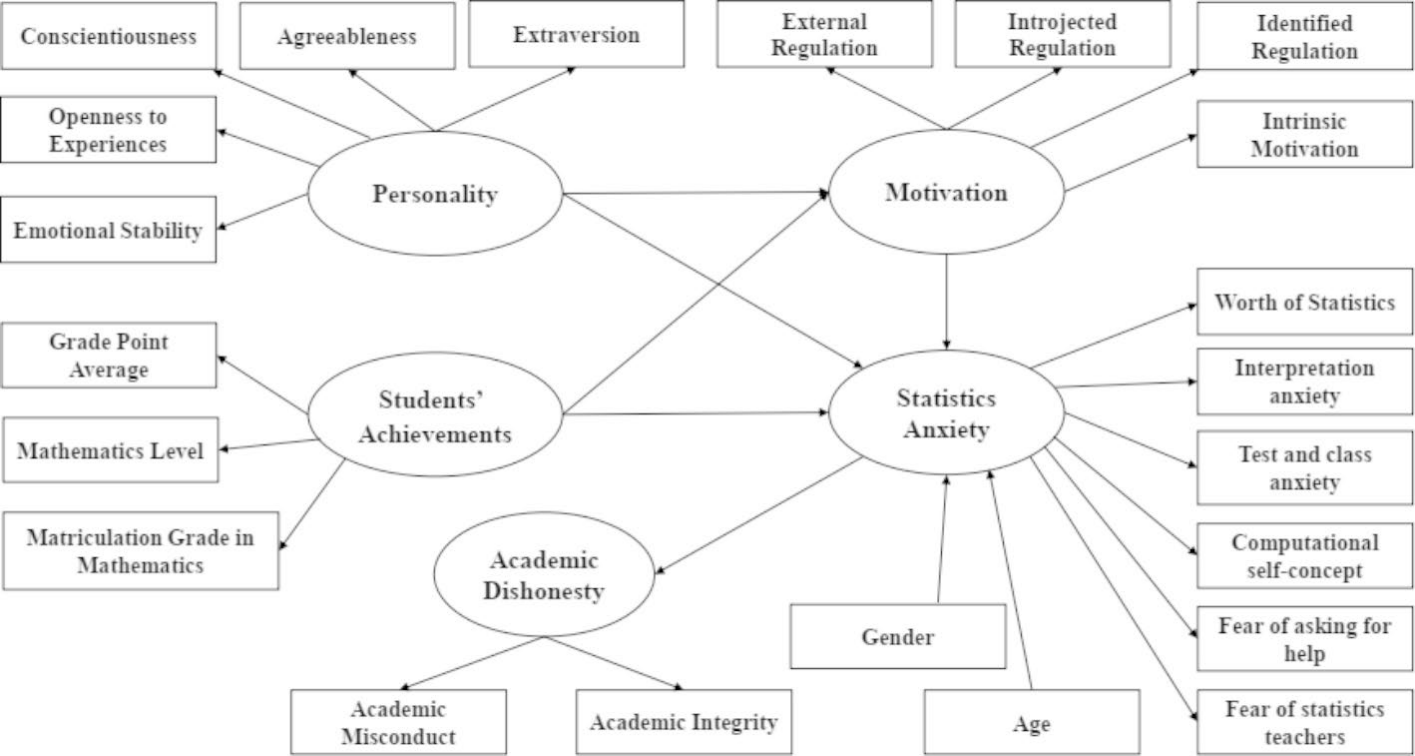
Structural Model for Determinants of Academic Dishonesty in Statistics Courses

Based on the literature above, the research model presents AD as assumed to be influenced by personality traits and students’ previous achievements with the mediation of motivation and SA (Fig. 1).

The research model presents personality traits (measured by extraversion, agreeableness, conscientiousness, openness to experiences, and emotional stability), students’ previous achievements (measured by mathematics level, grade point average, and matriculation grade in mathematics) with the mediation of the latent variable of motivation (measured by external regulation, introjected regulations, identifies regulation and intrinsic motivation), and SA (measured by worth of statistics, interpretation anxiety, test and class anxiety, computational self-concept, fear of asking for help, and fear of statistics teachers) as the factors assumed to influence AD..

## Methods

### Participants and procedure

Data were collected from five Israeli academic institutions students studying for bachelor’s degrees in the Social Sciences enrolled in introductory Statistics courses. There was a total of 649 participants, 7% were male and 93% female students. Mean age of participants was 23.5, ranging between 18 and 42, SD 7 years. Questionnaires were administered to the participants in three different course enrolment modalities through an online platform following the approval of the Ethics Committee. More than half of the students (59%) enrolled in POE, 18% in F2F, and 23% in ERT courses. The average time for filling out the questionnaires was 12 min. Fourteen per cent of the participants were excluded from the analysis as their survey instruments were incomplete (less than 80%) or carelessly completed. Among the participants, 6.5% reported high SA (the mean higher than 4 on a scale from 1 to 5). A significant difference was found between all the three learning environments [F_(2,646)_ = 36.637, p < 0.001] in SA (M = 2.50, SD = 0.60 for POE, M = 3.02, SD = 0.62 for F2F and M = 2.80, SD = 0.56 for ERT). Almost two-thirds of the participants (64.6%) reported having engaged in AD at least once in the POE learning environment, compared to 55% in the F2F and 43.5% in the ERT modality. A significant difference was found between all the three learning environments [F_(2,646)_ = 17.893, p < 0.001] in AD (M = 4.12, SD = 0.41 for POE, M = 3.85, SD = 0.44 for F2F and M = 3.99, SD = 0.41 for ERT)..

### Instruments

#### Dependent variables

Academic Dishonesty was measured directly through *the Academic Misconduct Scale* (Bolin 2004) and indirectly through *the Academic Integrity Inventory* (Kisamore et al. 2007). and validated these instruments to the Israeli context. The *Academic Misconduct Scale* comprises 10 items on a five-point Likert scale, in which 1 means “Never” and 5 “Many times”. Its reliability is excellent (0.91 Cronbach’s alpha). The *Academic Integrity Inventory* consists of 8 items on a five-point Likert scale, in which 1 means “Very unlikely” and 5 “Very likely”. Its reliability is acceptable (0.75 Cronbach’s alpha).

#### Mediating variables

Statistics Anxiety - This research uses the Hebrew version of the Statistics Anxiety Rating Scale (H-STARS), which is an abridged version of the STARS scale developed by Cruise et al. (1985). The H-STARS has been adapted to the Israeli context and found reliable and valid (Steinberger 2020). The Hebrew version of STARS comprises 30 items and employs six different subscales: worth of statistics; interpretation anxiety; test and class anxiety; computational self-concept; fear of asking for help; fear of statistics instructors. Participants answer questions about possible anxiety-inducing situations and their attitudes to statistics on a 5-point scale, in which 1 means no anxiety and 5 a great deal thereof. Steinberger (2020) has reported good internal consistency reliability (0.80–0.94). These are consistent with those presented previously in Cruise et al. (1985). Following the authors’ recommendation, calculating the overall score averages all questionnaire items, so the higher the score, the higher the anxiety level.

Motivational orientation – We employed *the Academic Self-Regulation Questionnaire (SRQ-A)* (Ryan and Connell 1989), which evaluates four types of motivation: intrinsic motivation, identified, introjected, and external regulation. Participants answered 17 questions employing a five-point Likert scale, in which 1 means “Not true at all and 5 “Very true”. As measured by Cronbach’s alpha, the questionnaire’s reliability is acceptable (0.75)..

#### Independent variables

Personality traits – The survey employs *the Ten Item Personality Inventory (TIPI)* scale by Gosling et al. (2003), which is comprised of 10 items developed to evaluate the personality traits of the participants on a five-point Likert scale, in which 1 means “Not true at all and 5 “Very true”. Two statements inform each trait. The reliability of this questionnaire, as measured by Cronbach’s alpha is questionable (0.63)..

Previous academic achievements are measured according to students’ high school mathematics level, grade point average, matriculation grade in mathematics, and course enrolment type..

### Plan of analysis

We have analysed the data through Structural Equation Modelling (SEM). Full information maximum likelihood estimates were computed using the Analysis of Moment Structures (AMOS) program (Arbuckle and Wothke 1999). The model was examined for the goodness of fit using χ2, comparative fit index (CFI), and root mean square error of approximation (RMSEA) fit indices. CFI values above 0.90 and 0.95 indicate adequate and good model fit, respectively, and RMSEA values below 0.08 and 0.05 indicate adequate and good model fit, respectively (Browne and Cudeck 1992; Hu and Bentler 1999). In addition, we used descriptive statistics and Pearson Correlations to analyse the data. Reliability analysis was done as well.

## Results

The descriptive statistics and correlations between the research variables are presented in Table 1.

**Table 1:** Descriptive Statistics and Inter-correlations among Variables

Variables	M	SD	1	2	3	4	5	6	7	8	9	10	11	12	13	14	15	16–17
1. Extraversion	3.37	0.81	**==**															
2. Agreeableness	3.84	0.71	0.083^*^	**==**														
3. Conscientiousness	4.11	0.71	0.107^**^	0.240^***^	**==**													
4. Openness to Experiences	3.73	0.74	0.248^***^	0.111^**^	0.226^***^	**==**												
5. Emotional Stability	3.63	0.83	0.169^***^	0.294^***^	0.311^***^	0.194^***^	**==**											
6. External Regulation	3.43	0.71	− 0.031	− 0.075^~^	0.005	0.002	− 0.132^**^	**0.54**										
7. Introjected Regulation	3.42	0.91	− 0.040	− 0.135^**^	0.121^**^	0.043	− 0.130^**^	0.505^***^	**0.72**									
8. Identified Regulation	4.18	0.92	− 0.029	− 0.037	0.217^***^	0.108^**^	0.015	0.229^***^	0.565^***^	**0.71**								
9. Intrinsic Motivation	3.16	1.12	− 0.029	−0.183^***^	0.154^***^	0.170^***^	0.059	0.276^***^	0.583^***^	0.608^***^	**0.79**							
10. Worth of Statistics	3.22	1.08	0.019	0.092^*^	− 0.071	−0.202^***^	−0.124^**^	− 0.004	−0.266^***^	−0.415^***^	−0.530^***^	**0.91**						
11. Interpretation anxiety	2.90	0.98	− 0.039	− 0.015	− 0.097^*^	− 0.221^***^	− 0.269^***^	0.140^***^	− 0.004	− 0.199^***^	− 0.256^***^	0.446^***^	**0.86**					
12. Test & class anxiety	3.01	1.04	− 0.042	− 0.046	− 0.123^**^	− 0.195^***^	− 0.320^***^	0.160^***^	0.051	− 0.143^***^	− 0.238^***^	0.472^***^	0.753^***^	**0.88**				
13. Computational self-concept	2.57	0.95	− 0.015	− 0.043	− 0.142^**^*	− 0.160^***^	− 0.296^***^	0.041	− 0.102^**^	− 0.308^***^	− 0.360^***^	0.656^***^	0.519^***^	0.591^***^	**0.86**			
14. Fear of asking for help	2.34	1.02	−0.099^*^	−0.158^***^	− 0.138^***^	− 0.233^***^	− 0.272^***^	0.159^***^	0.109^**^	− 0.126^**^	− 0.084^*^	0.293^***^	0.637^***^	0.673^***^	0.471^***^	**0.88**		
15. Fear of statistics teachers	2.50	0.86	− 0.017	−0.156^***^	− 0.153^***^	− 0.183^***^	− 0.319^***^	0.064	− 0.022	− 0.249^***^	− 0.283^***^	0.587^***^	0.496^***^	0.535^***^	0.708^***^	0.458^***^	**0.83**	
16. Academic Misconduct	2.68	0.62	− 0.073	−0.270^***^	− 0.047	0.015	− 0.152^***^	0.081^*^	0.128^**^	0.021	0.172^***^	0.117^**^	0.074^~^	− 0.046	− 0.044	0.073	0.036	**0.68**
17. Academic Integrity	4.75	0.48	−0.081^*^	−0.183^***^	− 0.213^***^	− 0.077^~^	− 0.151^***^	− 0.023	0.019	− 0.193^***^	− 0.028	0.023	0.131^**^	0.088^*^	0.130^**^	0.167^***^	0.153^***^	**0.92**

Notes: Reliability coefficients appear on the diagonal in bold. *p < 0.05; **p < 0.01; ***p < 0.001; n = 649

The results show significant negative correlations between all the five personality traits and AD. Furthermore, there is a significant negative correlation between identified regulation and AD and significant positive correlations between AD, external and introjected regulation, and intrinsic motivation. There are positive correlations between each of the components of SA and AD. Table 2 presents inter-correlations between statistics anxiety, motivation, personality traits, and the dependent variables of academic misconduct and academic integrity in the three learning environments..

**Table 2:** Descriptive statistics and inter-correlations between Statistics Anxiety and the research variables

	POE (n = 333)			Face-to-Face (n = 100)			ERT (n = 128)		
Variables	M	SD	r_p_	M	SD	r_p_	M	SD	r_p_
Extraversion	3.42	0.85	0.003	3.44	0.83	− 0.105	3.22	0.78	− 0.118
Agreeableness	4.05	0.66	− 0.045	3.60	0.70	− 0.080	3.61	0.69	− 0.151^*^
Conscientiousness	4.05	0.70	− 0.085	4.10	0.69	− 0.134	4.26	0.70	− 0.225^**^
Openness to Experiences	3.69	0.72	− 0.127^*^	3.81	0.82	− 0.408^***^	3.78	0.68	− 0.332^***^
Emotional Stability	3.69	0.84	− 0.249^***^	3.55	0.85	− 0.489^***^	3.53	0.83	− 0.402^***^
External Regulation	3.24	0.59	0.165^**^	3.49	0.70	0.147	3.50	0.80	0.151^*^
Introjected Regulation	3.04	0.87	− 0.037	3.67	0.76	0.166	3.72	0.75	− 0.065
Identified Regulation	3.88	0.99	− 0.295^***^	4.41	0.79	− 0.304^**^	4.50	0.69	− 0.306^***^
Intrinsic Motivation	2.57	0.91	− 0.388^***^	3.72	1.05	− 0.348^***^	3.64	1.02	− 0.423^***^
Statistics Anxiety	2.84	0.71	==	2.81	0.89	==	2.72	0.87	==
Academic Misconduct	2.50	0.60	− 0.103	3.02	0.62	0.044	2.80	0.56	0.064
Academic Integrity	4.74	0.45	0.167^**^	4.72	0.53	0.014	4.79	0.50	0.163^*^

Results show significant negative correlations between the personality traits of openness to experience and emotional stability and statistics anxiety in all three learning environments, as well as between the personality traits of agreeableness and conscientiousness and statistics anxiety, albeit in the ERT sample only. In addition, there is a significant positive correlation between external regulation and statistics anxiety in the POE and ERT learning environments and significant negative correlations between identified regulation and intrinsic motivation and statistics anxiety in all three learning environments. Nonetheless, significant positive correlations between statistics anxiety and dependent variables were found in the POE and ERT samples, though not in the F2F one. The AD variable was modelled by the variables of academic misconduct and academic integrity, by the latent variable of personality, and those of motivation, and of students’ previous achievements with the mediation of the latent variable of SA. The data fit the academic dishonesty model marginally well (χ2 = 1,426.37, N = 649, df = 564, p < 0.001, CFI = 0.801, RMSEA = 0.049)..

### Academic dishonesty analysis - POE sample

**Fig. 2:**
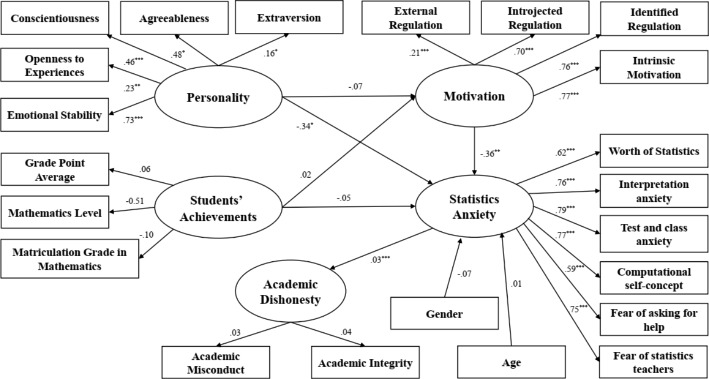
Structural model for determinants of Academic Dishonesty - POE Sample

The structural model of academic dishonesty in the POE sample is illustrated in Fig. 2.

The results of the analysis indicate that the variance in AD is explained by students’ personality traits with the mediation of SA. Accordingly, the POE sample supports H_2_. SA is the variable having a greater impact on academic misconduct with a total effect of 67%. As shown in Fig. 2, test and class anxiety are among the most influential components of SA. It has one of the highest effects (*b* = 0.79, *p* < 0.001), meaning that the higher a student’s level of SA as to test and class anxiety, the higher their propensity to cheat. The same applies to the component of SA regarding computational self-concept. It has been found to have a strong significant effect as well (*b* = 0.77, *p* < 0.001), while the higher the student’s [level of SA regarding] computational self-concept, the higher the probability that they engage in academic misconduct. Interpretation anxiety (*b* = 0.76, *p* < 0.001) is a further strong factor influencing academic misconduct. Accordingly, SA regarding interpretation anxiety increases academic misconduct. In addition, personality traits were found to have a significant negative impact on SA (*b*= -0.34, *p* < 0.05). All of the five personality traits have a significant effect on the mediating variable of SA: Extraversion *(b* = 0.16, *p* < 0.05), agreeableness (*b* = 0.48, *p* < 0.05), conscientiousness (*b* = 0.46, *p* < 0.001), openness to experience (*b* = 0.23, *p* < 0.01) and emotional stability (*b* = 0.73, *p* < 0.001). Accordingly, the higher levels of a student’s personality traits, the less anxious they is. Motivation was also found to have a negative significant impact on SA (*b*= -0.36, *p* < 0.01), while all motivation types have a significant effect on the mediating variable: external regulation (*b* = 0.21, *p* < 0.001), introjected regulation (*b* = 0.70, *p* < 0.001), identified regulation (*b* = 0.76 *p* < 0.001) and intrinsic motivation (*b* = 0.77, *p* < 0.001). In other words, the higher the student’s motivation, the lower they level of SA is.

### Academic dishonesty analysis - F2F sample

**Fig. 3:**
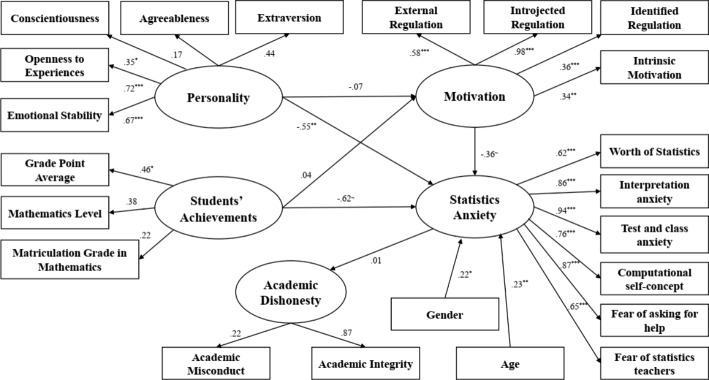
Structural model for determinants of Academic Misconduct – F2F Sample

The structural model of AD in the F2F sample is illustrated in Figure 3.

The analysis’s results indicate that the variance in AD is explained by students’ personality traits and motivation, with no significant effect of SA as a mediator. Therefore no support for the four hypotheses was obtained in the F2F sample. As shown in Fig. 3, personality traits were found to have a negative significant impact on SA (b= -0.55, p < 0.01), while three personality traits have a significant effect on the mediating variable: conscientiousness (b = 0.35, p < 0.05), openness to experience (b = 0.72, p < 0.001) and emotional stability (b = 0.67, p < 0.001). This means that the higher a student’s personality traits, the lower they level of SA is. Motivation was also found to have a negatively marginal significant impact on SA (b= -0.36, p = 0.065), while all motivation types have a significant effect on the mediating variable: external regulation (b = 0.58, p < 0.001), introjected regulation (b = 0.98, p < 0.001), identified regulation (b = 0.36 p < 0.001) and intrinsic motivation (b = 0.34, p < 0.01). In other words, the higher a student’s motivation, the less anxious they is. Grade point average (b = 0.46, p < 0.05) is a further variable having a significant negative effect on SA. The higher a student’s grade point average, the lower they SA is. Gender and age were also found to have a significant effect on SA (b = 0.22, p < 0.05 and b = 0.23, p < 0.01, respectively). Accordingly, women experience greater SA than their male counterparts, and the older the students age, the greater the SA.

### Academic dishonesty analysis - ERT sample

**Fig. 4:**
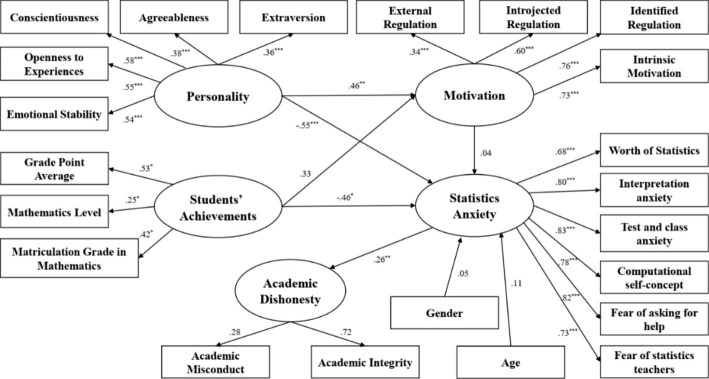
Structural model for determinants of Academic Misconduct - ERT Sample

The structural model of AD in the ERT sample is illustrated in Fig. 4.

The analysis’s results indicate that the variance in AD is explained by students’ personality traits and students’ previous achievement, with the mediation of SA. Therefore, H_2_ and H_4_ were confirmed in the ERT sample. SA is the variable having the greatest impact on academic misconduct, with a total effect of 49%. As shown in Fig. 4, test and class anxiety are among the most influential components of SA; it has one of the higher effects (b = 0.83, p < 0.001). The higher a student’s level of SA as to test and class anxiety, the higher their propensity to cheat. Similarly, the component of SA regarding fear of asking for help was also found to have a strong significant effect (b = 0.82, p < 0.001). The higher a student’s level of SA as to fear of asking for help, the higher the probability they engage in academic misconduct. In addition, interpretation anxiety (b = 0.80, p < 0.001) is a further strong factor influencing academic misconduct. Moreover, personality traits were found to have a negative significant impact on SA (b= -0.55, p < 0.001), along with a positive significant effect on motivation (b = 0.46, p < 0.01), while all the personality traits have significant effect on the mediating variables: extraversion (b = 0.36, p < 0.001), agreeableness (b = 0.38, p < 0.001), conscientiousness (b = 0.58, p < 0.001), openness to experience (b = 0.55, p < 0.001) and emotional stability (b = 0.54, p < 0.001). This means that the higher a student’s levels of one of the above personality traits, the more motivated and less anxious they is. Another set of variables having a negative significant effect on SA are those related to previous student achievements (b= -0.46, p < 0.05): grade point average (b = 0.53, p < 0.05), mathematics level (b = 0.25, p < 0.05) and matriculation grade in mathematics (b = 0.42, p < 0.05). The higher one’s previous student achievements are, the lower they level of SA.

Table 3 summarizes the testing results for the research hypotheses.

**Table 3:** Hypotheses testing results

Course Type	Constructs	H	Β	SE	CR	p-value	Support
POE	Statistics Anxiety→ Academic Dishonesty		− 0.03	0.05	-3.629	^***^	Yes
	Personality → Statistics Anxiety		− 0.34	0.70	-2.157	0.03^*^	Yes
	Motivation → Statistics Anxiety		− 0.36	0.63	-2.88	^**^	Yes
	Personality → Motivation → Statistics Anxiety	H_1_	0.04	(-0.047; 0.583)		0.262	No
	Personality → Statistics Anxiety → Academic Dishonesty	H_2_	1.13^1^	(0.022; 2.722)		0.010^*^	Yes
	Students’ Achievement → Statistics Anxiety		0.22	0.07	-0.145	0.884	No
	Students’ Achievement → Motivation → Statistics Anxiety	H_3_	− 0.05	(-0.677; − 0.005)		0.034^*^	Yes
	Students’ Achievement → Statistics Anxiety → Academic Dishonesty	H_4_	0.16	(-0.118; 0.433)		0.833	No
F2F	Statistics Anxiety→ Academic Dishonesty		0.10	0.08	0.211	0.833	No
	Personality → Statistics Anxiety		− 0.55	0.35	-3.113	0.002	Yes
	Motivation → Statistics Anxiety		− 0.36	0.21	1.847	0.065^~^	Yes
	Personality → Motivation → Statistics Anxiety	H_1_	− 0.019	(-0.333; 0.109)		0.548	No
	Personality → Statistics Anxiety → Academic Dishonesty	H_2_	− 0.02	(-0.068; 0.645)		0.128	No
	Students’ Achievement → Statistics Anxiety		− 0.62	1.01	-1.892	0.059^~^	Yes
	Students’ Achievement → Motivation → Statistics Anxiety	H_3_	0.037	(-0.417; 1.223)		0.869	No
	Students’ Achievement → Statistics Anxiety → Academic Dishonesty	H_4_	− 0.03	(-0.137; 924)		0.119	No
ERT	Statistics Anxiety→ Academic Dishonesty		− 0.26	0.05	-2.659	0.008	Yes
	Personality → Statistics Anxiety		− 0.55	0.44	-3.332	^***^	Yes
	Motivation → Statistics Anxiety		0.04	0.34	0.273	0.785	No
	Personality → Motivation → Statistics Anxiety	H_1_	− 0.04	(-0.808; 1.309)		0.850	No
	Personality → Statistical Anxiety → Academic Dishonesty	H_2_	0.51	(0.143; 1.207)		0.01^*^	Yes
	Students’ Achievement → Statistics Anxiety		− 0.46	0.85	-2.078	0.038^*^	Yes
	Students’ Achievement → Motivation → Statistics Anxiety	H_3_	− 0.04	(-0.948; 2.015)		0.850	No
	Students’ Achievement → Statistics Anxiety → Academic Dishonesty	H_4_	0.66	(0.223; 2.841)		0.02^*^	Yes

Notes: β = standardized regression weight; SE, standardized error; CR, critical ratio. *p < 0.05; **p < 0.01, ***p < 0.001

The confidence interval of 95% in Brackets

^1^We conducted a Multicollinearity analysis, which showed that there is no multicollinearity between the research variables: VIF of all the research variables ranged from 1.048 to 2.730 (less than 5) and Tolerance ranged from 0.366 to 0.954 (above 0.2)

As shown in Table 3, the analysis results indicate that there was no significant indirect effect between personality traits and SA through the mediation of motivation in any of the learning environments. Accordingly, no support for H_1_ was obtained. A significant indirect effect between personality traits and AD mediated by SA was found in the POE and the ERT samples, thus partially confirming H_2_. A significant indirect effect between students’ achievements and SA through the mediation of motivation was found only in the POE sample, thus partially confirming H_3_. A significant indirect effect between students’ achievements and AD through the mediation of SA was found only in the ERT sample, thus partially confirming H_4_..

As shown in Table 4, the results of the multi-group analysis indicate that there is a significant difference between all course types: POE, F2F, and ERT, thus confirming H_4_.

**Table 4:** Presents a comparison among the learning environments

Course Type	NFI Delta-1	DF	*p*-value	Difference
POE vs. F2F	0.107	43	^***^	Yes
POE vs. ERT	0.168	43	^***^	Yes
F2F vs. ERT	0.023	43	0.016	Yes
General Model	0.171	86	^***^	Yes

## Discussion

Academic institutions’ promotion and maintenance of academic integrity are significant concerns (Chugh et al. 2021). The same applies to digital integrity, which has become a crucial 21st-century skill impacting students’ ability to handle and share knowledge ethically (Miller 2019) while maintaining their performance levels (Amigud and Lancaster 2019). In this context, the present research presents for the first time a comparison between academic ethical behaviour, SA, personality traits, and motivation in different learning environments (F2F, POE & ERT). In addition, it relies on Self-Determination Theory and expands the existing literature on students’ dishonest behaviour (lack of academic integrity) and their motivations for engaging in this in statistics introductory courses. In line with the scholarly literature (Krou et al. 2021), we believe that understanding the motivational and anxiety-related mechanisms involved in unethical academic behaviours is key to designing future teaching, learning, and assessment approaches (Etgar et al. 2019; Steinberger et al. 2021).

The results show that learning environments (F2F, POE & ERT) affect and play a significant role in interacting with SA, motivation, personality traits, and AD (H_4_). Moreover, findings show that AD is more prevalent in POE than in F2F and ERT environments. This study’s findings improve the model employed in previous studies (Peled et al. 2019; Steinberger et al. 2021) by revealing that SA plays a significant role alongside the circumstances comprising learning environments mediating between personality traits and AD. More concretely, this study shows that learning environments determine the mediating role of SA. In digital learning environments (POE, ERT), mediation has been found between students’ personality traits and AD. No similar parallel mediation could be established in the physical learning environment, F2F. In line with the scholarly literature (Whittle et al. 2020), these differences may be attributed to the impacts an instructor’s presence has in different learning environments. This difference may be due to the virtual communication and lack of physical presence of academic instructors in both POE and ERT learning modalities, which may increase students’ anxiety. However, F2F learning is mostly characterized by a student’s direct and immediate interaction with the instructor and fellow students. The lack of a teacher’s physical presence may lead to uncertainty and anxiety and directly impact students’ ethical disinhibition.

Additionally, the differences from examining the two digital environments show that in the ERT one, SA mediates between students’ previous achievements and AD. No similar parallel mediation could be found in the POE environment. This difference may be attributed to educational delivery methods (asynchronous vs. synchronous), which affect learning quality and process (Steinberger et al. 2021). Furthermore, the immediate necessity to move to digital learning without prior preparation during the global pandemic has led students to severe distress. These have been compelled to deal with existential health anxiety and a state of ongoing uncertainty while continuing to take a demanding course, potentially awakening SA. In addition, the quality of distance teaching is lower in ERT due to being imposed at once without any prior pedagogical preparation (Hollweck and Doucet 2020). Accordingly, students facing exceptional and extreme situations like this may rely exclusively on their previous academic experience or achievements in studies in general and, more concretely, in mathematics. A successful student may be less anxious about statistics, thus refraining from unethical behaviour. On the other hand, in asynchronous online courses, one’s experience of previous academic success is not related to AD as mediated by SA.

## Conclusion

Learning is a socially determined activity (Goodhart 2020), as individuals learn from and with others, even at a distance. Hence, online courses should be designed according to student-centred approaches (Rapanta et al. 2020). The foregoing may include: Instructor’s immediacy, improved communication, pre-planned real-life based on learning tasks (Neumann et al. 2013), and monitoring of student progress, for which using continuous formative assessment is key (Torres Martín et al. 2021). Such an approach may create an optimal class climate, overcome the limitations of digital learning, and decrease SA and AD. For, instructors’ immediacy and direct communication are concrete in F2F instruction, POE and ERT rely on electronically mediated communication. This, in turn, promotes students’ sense of self-competence and autonomy throughout their learning processes, thus reducing dishonesty (Kanat-Maymon et al. 2015). Additionally, positive attitudes towards learning statistics are crucial to motivate students and awaken their interest in the subject. These significantly impact the general class climate and student academic performance (Bromage et al. 2021). In this context, recent scholarship has revealed an increasing trend among students to pay external agents to prepare their academic assignments (contract cheating) (Birks et al. 2020). The major causes for this are students’ dissatisfaction with teaching and learning environments, the stress of time, and the perception of cheating opportunities resulting from the current variety of technological possibilities facilitating non-ethical behaviour (Amzalag et al. 2021). Scholars have consequently stressed that deepening student engagement and learning requires that part-time faculty take part in discussing and communicating ideas and creating clear policies and shared tasks (Artiukhov and Liuta 2017).

### Limitations and future research

Data were collected before academic institutions formulated clear examination policies to transition to distance learning. Hence, respondents experienced ambiguity regarding the course’s evaluation method (test or paper) and could not design unethical behaviour strategies. Nor could they know whether the latter would take place on campuses or be carried out remotely electronically. Like any other empirical model, the present model is a specific theoretical construct analysing and reflecting a given practice (its data). In other words, our model offers a particularized theoretical perspective of a general socio-cultural phenomenon. This entails that research, theory, and practice could all potentially benefit from similar tests focusing on additional contexts and employing other predictors. Future research may investigate the impact of hybrid learning environments on the relationship comprising SA and AD.

## Data Availability

Not applicable.

## Abbreviations

**AD**: Academic Dishonesty.
**ERT**: Emergency Remote Teaching.
**F2F**: Face To Face.
**FFM**: Five-Factor Model.
**POE**: Planned Online Environment.
**SA**: Statistics Anxiety.
**SDT**: Self-Determination Theory.

## References

[CR1] Agbaria Q, Mokh AA (2021) Coping with stress during the coronavirus outbreak: The contribution of big five personality traits and social support.Int J Ment Health Addict1–19 10.1007/s11469-021-00486-2 PMC7819145 33500687

[CR2] Amigud A, Lancaster T (2019). 246 reasons to cheat: An analysis of students’ reasons for seeking to outsource academic work. Comput Educ.

[CR3] Amzalag M, Shapira N, Dolev N (2021) Two sides of the coin: Lack of academic integrity in exams during the Corona Pandemic, students’ and lecturers’ perceptions.J Acad Ethics1–21 10.1007/s10805-021-09413-5 PMC8027972 33846681

[CR4] Arbuckle JL, Wothke W (1999). Amos 4.0 User’s Guide.

[CR5] Armellini A, Antunes VT, Howe R (2021). Student perspectives on learning experiences in a higher education active blended learning context. TechTrends.

[CR6] Arniatika S (2020). Personality traits, motivational 0rientations and speaking achievement in the EFL context. Int J Indones Educ Teach.

[CR7] Artiukhov AY, Liuta OV (2017). Academic integrity in Ukrainian higher education: Values, skills, actions. Bus Ethics Leadersh.

[CR8] Bacon AM, McDaid C, Williams N, Corr PJ (2020). What motivates academic dishonesty in students? A reinforcement sensitivity theory explanation. Br J Educ Psychol.

[CR9] Badiozaman IFA (2021). Exploring online readiness in the context of the COVID 19 pandemic. Teach High Educ.

[CR10] Becerra BLG, Almendra MPR (2020) Measuring student motivation in a statistics course supported by podcast using Reduced Instructional Materials Motivation Survey (RIMMS). In: 2020 X International Conference on Virtual Campus (JICV). IEEE, pp 1–4

[CR11] Berndt M, Schmidt FM, Sailer M (2021). Investigating statistical literacy and scientific reasoning & argumentation in medical-, social sciences-, and economics students. Learn Individ Differ.

[CR12] Birks M, Mills J, Allen S, Tee S (2020). Managing the mutations: academic misconduct Australia, New Zealand, and the UK. Int J Educ Integr.

[CR13] Bolin AU (2004). Self-control, perceived opportunity, and attitudes as predictors of academic dishonesty. J Psychol.

[CR14] Bromage A, Pierce S, Reader T, Compton L (2021). Teaching statistics to non-specialists: challenges and strategies for success. J Furth High Educ.

[CR15] Browne MW, Cudeck R (1992). Alternative ways of assessing model fit. Sociol Methods Res.

[CR16] Butler-Henderson K, Crawford J (2020). A systematic review of online examinations: A pedagogical innovation for scalable authentication and integrity. Comput Educ.

[CR17] Chew KHP, Dillon DB (2014). Statistics anxiety and the Big Five personality factors. Procedia-Social Behav Sci.

[CR18] Chiang F, Zhu D, Yu W (2022) A systematic review of academic dishonesty in online learning environments. J Comput Assist Learn 1–22. 10.1111/jcal.12656

[CR19] Christensen Hughes J, Eaton SE, Eaton SE, Hughes JC (2022). Student integrity violations in the academy: More than a decade of growing complexity and concern. Academic Integrity in Canada: An Enduring and Essential Challenge.

[CR20] Chugh R, Luck JA, Turnbull D, Pember ER (eds) (2021) Back to the classroom: Educating sessional teaching staff about academic integrity. J Acad Ethics 19:115–134

[CR21] Crawford J (2020). COVID-19: 20 countries’ higher education intra-period digital pedagogy responses. J Appl Learn Teach.

[CR22] Cruise RJ, Cash RW, Bolton DL (1985) Development and validation of an instrument to measure statistical anxiety. In: American Statistical Association Proceedings of the Section on Statistical Education. pp 92–97

[CR23] Cui S, Zhang J, Guan D (2019). Antecedents of statistics anxiety: An integrated account. Pers Individ Dif.

[CR24] Deci EL, Ryan RM (2008) Self-Determination Theory: A Macrotheory of Human Motivation, Development, and Health. 10.1037/a0012801

[CR25] Dimitriadis E, Anastasiades T, Karagiannidou D, Lagaki M (2017). Creativity and entrepreneurship: The role of gender and personality. Int J Bus Econ Sci Appl Res.

[CR26] du Rocher AR (2020). Active learning strategies and academic self-efficacy relate to both attentional control and attitudes towards plagiarism. Act Learn High Educ.

[CR27] Elsalem L, Al-Azzam N, Jum’ah AA, Obeidat N (2021). Remote E-exams during Covid-19 pandemic: A cross-sectional study of students’ preferences and academic dishonesty in faculties of medical sciences. Ann Med Surg.

[CR28] Erguvan ID (2021). The rise of contract cheating during the COVID-19 pandemic: a qualitative study through the eyes of academics in Kuwait. Lang Test Asia.

[CR29] Eshet Y, Grinautski K, Peled Y, Barczyk C (2014). No more excuses: Personality traits and academic dishonesty in online courses. J Stat Sci Appl.

[CR30] Etgar S, Blau I, Eshet-Alkalai Y (2019). White-collar crime in academia: Trends in digital academic dishonesty over time and their effect on penalty severity. Comput Educ.

[CR31] Faber G, Drexler H (2019). Predicting education science students’ Statistics Anxiety: The role of prior experiences within a framework of domain-specific motivation constructs. High Learn Res Commun.

[CR32] Foutz B, Violanti M, Kelly S, Prentiss SM (2021). Teacher immediacy behaviors and students’ public speaking anxiety: More and less helpful than anticipated. Basic Commun Course Annu.

[CR33] Gamage KAA, de Silva EK, Gunawardhana N (2020). Online delivery and assessment during Covid-19: Safeguarding academic integrity. Educ Sci.

[CR34] Giluk TL, Postlethwaite BE (2015). Big Five personality and academic dishonesty: A meta-analytic review. Pers Individ Dif.

[CR35] Goodhart C (2020). Learning is a social activity. Rev Behav Financ.

[CR36] Gosling SD, Rentfrow PJ, Swann WB (2003). A very brief measure of the Big-Five personality domains. J Res Pers.

[CR37] Greenberg DN, Clair JA, Maclean TL (2007). Enacting the role of management professor: Lessons from Athena, Prometheus, and Asclepius. Acad Manag Learn Educ.

[CR38] Guerrero-Dib JG, Portales L, Heredia-Escorza Y (2020). Impact of academic integrity on workplace ethical behaviour. Int J Educ Integr.

[CR39] Helms JL (2014). Comparing student performance in online and face-to-face delivery modalities. J asynchronous Learn networks.

[CR40] Hensley LC, Kirkpatrick KM, Burgoon JM (2013). Relation of gender, course enrollment, and grades to distinct forms of academic dishonesty. Teach High Educ.

[CR41] Hodges C, Moore S, Lockee B (2020). The difference between emergency remote teaching and online learning. Educ Rev.

[CR42] Hollweck T, Doucet A (2020) Pracademics in the pandemic: pedagogies and professionalism. J Prof Cap community

[CR43] Hu L, Bentler PM (1999). Cutoff criteria for fit indexes in covariance structure analysis: Conventional criteria versus new alternatives. Struct Equ Model.

[CR44] Ikram F, Rabbani MA (2021) Academic integrity in traditional vs online undergraduate medical education amidst Covid-19 pandemic.Cureus13 10.7759/cureus.13911 PMC8051425 33880266

[CR45] Kanat-Maymon Y, Benjamin M, Stavsky A (2015). The role of basic need fulfillment in academic dishonesty: A self-determination theory perspective. Contemp Educ Psychol.

[CR46] Kisamore JL, Stone TH, Jawahar IM (2007). Academic integrity: The relationship between individual and situational factors on misconduct contemplations. J Bus Ethics.

[CR47] Koscielniak M, Bojanowska A (2019). The role of personal values and student achievement in academic dishonesty. Front Psychol.

[CR48] Krou MR, Fong CJ, Hoff MA (2021). Achievement motivation and academic dishonesty: A meta-analytic investigation. Educ Psychol Rev.

[CR49] Kudeikina I, Mihailovs IJ, Zīvarts J (2022). Academic integrity in education in the context of sustainable development of society. Eur J Sustain Dev.

[CR50] Lavasani MG, Weisani M, Shariati F (2014). The role of achievement goals, academic motivation in statistics anxiety: Testing a causal model. Procedia - Soc Behav Sci.

[CR51] Lee SD, Kuncel NR, Gau J (2020). Personality, attitude, and demographic correlates of Academic Dishonesty: A meta-analysis. Psychol Bull.

[CR52] Levpušček MP, Cukon M (2020). That old devil called ‘Statistics’: Statistics Anxiety in University Students and related factors. Cent Educ Policy Stud J.

[CR53] Li C, Lalani F (2020) The COVID-19 pandemic has changed education forever. This is how. In: World Econ. forum. https://www.weforum.org/agenda/2020/04/coronavirus-education-global-covid19-online-digital-learning/

[CR54] Locquiao J, Ives B (2020). First-year university students’ knowledge of academic misconduct and the association between goals for attending university and receptiveness to intervention. Int J Educ Integr.

[CR55] Lowenthal P, Borup J, West R, Archambault L (2020). Thinking beyond Zoom: Using asynchronous video to maintain connection and engagement during the COVID-19 Pandemic. J Technol Teach Educ.

[CR56] Luo Z, Subramaniam G, O’Steen B (2020). Will anxiety boost motivation? The relationship between anxiety and motivation in foreign language learning. Malaysian J ELT Res.

[CR57] Malesky A, Grist C, Poovey K, Dennis N (2022). The effects of peer influence, honor codes, and personality traits on cheating behavior in a university setting. Ethics Behav.

[CR58] Maqableh M, Alia M (2021). Evaluation online learning of undergraduate students under lockdown amidst COVID-19 Pandemic: The online learning experience and students’ satisfaction. Child Youth Serv Rev.

[CR59] McCabe D, Butterfield KD, Treviño K (2006). Academic Dishonesty in Graduate Business Programs. Acad Manag Learn Educ.

[CR60] McCrae RR, Costa PT (1987). Validation of the Five-Factor Model of personality across instruments and observers. J Pers Soc Psychol.

[CR61] Meiring H (2019) Unethical decision making: towards understanding the factors that influence a white collar criminal’s decision to commit a crime. University of Pretoria

[CR62] Mesny A, Rivas DP, De Haro SP (2021). Business school professors’ teaching approaches and how they change. Acad Manag Learn Educ.

[CR63] Miller L, Power R (2019). Digital integrity as a 21st century skill. Technology and the Curriculum: Summer 2019.

[CR64] Mørk G, Magne TA, Carstensen T et al (2020) Associations between learning environment variables and students’ approaches to studying: A cross-sectional study. BMC Med Educ 20. 10.1186/s12909-020-02033-4 10.1186/s12909-020-02033-4 PMC7171764 32312267

[CR65] Müller FH, Palekčić M, Beck M, Wanninger S (2006). Personality, motives and learning environment as predictors of self-determined learning motivation. Rev Psychol.

[CR66] Muntada CI (2013). Personality, procrastimation and cheating in students from different university degree programs. Electron J Res Educ Psychol.

[CR67] Murtonen M (2015). University students’ understanding of the concepts empirical, theoretical, qualitative and quantitative research. Teach High Educ.

[CR68] Neumann DL, Hood M, Neumann MM (2013). Using real-life data when teaching statistics: Student perceptions of this strategy in an introductory statistics course. Stat Educ Res J.

[CR69] O’Bryant M, Natesan Batley P, Onwuegbuzie AJ (2021). Validation of an adapted version of the Statistical Anxiety Scale in english and its relationship to attitudes toward statistics. SAGE Open.

[CR70] Onwuegbuzie AJ, Wilson VA (2003). Statistics Anxiety: Nature, etiology, antecedents, effects, and treatments-a comprehensive review of the literature. Teach High Educ.

[CR71] Ozoliņa R, Bēriņa LH (2021). Academic integrity in Latvia’s higher education institutions. SSE Riga Student Res Pap.

[CR72] Parnther C (2020). Academic misconduct in higher education: A comprehensive review. J High Educ Policy Leadersh Stud.

[CR73] Peiró-Signes Á, Trull O, Segarra-Oña M, García-Díaz JC (2021). Anxiety towards statistics and its relationship with students’ attitudes and learning approach. Behav Sci (Basel).

[CR74] Peled Y, Eshet Y, Barczyk C, Grinautski K (2019). Predictors of Academic Dishonesty among undergraduate students in online and face-to-face courses. Comput Educ.

[CR75] Rapanta C, Botturi L, Goodyear P (2020). Online university teaching during and after the Covid-19 crisis: Refocusing teacher presence and learning activity. Postdigital Sci Educ.

[CR76] Reedy A, Pfitzner D, Rook L, Ellis L (2021). Responding to the COVID-19 emergency: student and academic staff perceptions of academic integrity in the transition to online exams at three Australian universities. Int J Educ Integr.

[CR77] Roe J (2022). Reconceptualizing academic dishonesty as a struggle for intersubjective recognition: a new theoretical model. Humanit Soc Sci Commun 2022.

[CR78] Ryan RM, Connell JP (1989) Self-regulation questionnaire. Unpubl. Manuscr

[CR79] Ryan RM, Deci EL (2020). Intrinsic and extrinsic motivation from a self-determination theory perspective: Definitions, theory, practices, and future directions. Contemp Educ Psychol.

[CR80] Sefcik L, Striepe M, Yorke J (2020). Mapping the landscape of academic integrity education programs: what approaches are effective?. Assess Eval High Educ.

[CR81] Shi Y, Tong M, Long T (2021). Investigating relationships among blended synchronous learning environments, students’ motivation, and cognitive engagement: A mixed methods study. Comput Educ.

[CR82] Siew CSQ, McCartney MJ, Vitevitch MS (2019). Using network science to understand statistics anxiety among college students. Scholarsh Teach Learn Psychol.

[CR83] Sleep CE, Lynam DR, Miller JD (2021). A comparison of the validity of very brief measures of the Big Five/Five-Factor Model of personality. Assessment.

[CR84] Sorge C, Schau C (2002) Impact of engineering students’ attitudes on achievement in statistics: A structural model. In: annual meeting of the American Educational Research Association. New Orleans. Citeseer

[CR85] Steinberger P (2020). Assessing the Statistical Anxiety Rating Scale as applied to prospective teachers in an Israeli Teacher-Training College. Stud Educ Eval.

[CR86] Steinberger P, Eshet Y, Grinautsky K (2021). No anxious student is left behind: Statistics anxiety, personality traits, and academic dishonesty—lessons from covid-19. Sustain.

[CR87] Tindall IK, Fu KW, Tremayne K, Curtis GJ (2021). Can negative emotions increase students’ plagiarism and cheating?. Int J Educ Integr.

[CR88] Tonguç G, Ozaydın Ozkara B (2020). Automatic recognition of student emotions from facial expressions during a lecture. Comput Educ.

[CR89] Torres Martín C, Acal C, El Honrani M, Mingorance Estrada ÁC (2021). Impact on the Virtual Learning Environment Due to COVID-19. Sustainability.

[CR90] Trassi AP, Leonard SJ, Rodrigues LD et al (2022) Mediating factors of statistics anxiety in university students: a systematic review and meta-analysis. Ann N Y Acad Sci 10.1111/nyas.14746 35211989

[CR91] Turnbull D, Chugh R, Luck J (2021) Transitioning to E-Learning during the COVID-19 pandemic: How have Higher Education Institutions responded to the challenge?Educ Inf Technol1–19 10.1007/s10639-021-10633-w PMC8220880 34177349

[CR92] Turner KL, Adams JD, Eaton SE (2022) Academic integrity, STEM education, and COVID-19: A call to action. Cult Stud Sci Educ 1–9. 10.1007/s11422-021-10090-4 10.1007/s11422-021-10090-4 PMC8881977 35251362

[CR93] Valtonen T, Leppänen U, Hyypiä M (2021). Learning environments preferred by university students: A shift toward informal and flexible learning environments. Learn Environ Res.

[CR94] Vaziri S, Vaziri B, Novoa LJ, Torabi E (2022). Academic motivation in introductory business analytics courses: A bayesian approach. INFORMS Trans Educ.

[CR95] Venn E, Park J, Andersen LP, Hejmadi M (2020) How do learning technologies impact on undergraduates’ emotional and cognitive engagement with their learning? Teach High Educ 1–18. 10.1080/13562517.2020.1863349

[CR96] Walsh LL, Lichti DA, Zambrano-Varghese CM (2021). Why and how science students in the United States think their peers cheat more frequently online: Perspectives during the COVID-19 pandemic. Int J Educ Integr.

[CR97] Whalen J (2020). Should teachers be trained in Emergency Remote Teaching? Lessons learned from the Covid-19 Pandemic. J Technol Teach Educ.

[CR98] Whittle C, Tiwari S, Yan S, Williams J (2020). Emergency remote teaching environment: A conceptual framework for responsive online teaching in crises. Inf Learn Sci.

[CR99] Zalts R, Green N, Tackett S, Lubin R (2021). The association between medical students’ motivation with learning environment, perceived academic rank, and burnout. Int J Med Educ.

[CR100] Zhang H, Shi Y, Zhou ZE (2020). Good people do bad things: How anxiety promotes unethical behavior through intuitive and automatic processing. Curr Psychol.

